# A series of helical α-synuclein fibril polymorphs are populated in the presence of lipid vesicles

**DOI:** 10.1038/s41531-020-00122-1

**Published:** 2020-08-19

**Authors:** Richard M. Meade, Robert J. Williams, Jody M. Mason

**Affiliations:** Department of Biology and Biochemistry, University of Bath, Claverton Down, Bath, BA2 7AY UK

**Keywords:** Proteins, Cellular neuroscience

## Abstract

α-Synuclein (αS) deposition is a defining characteristic of Parkinson’s disease (PD) pathology, and other synucleinopathies. αS aggregates in disease, leading to the generation of neuronal inclusions known as Lewy bodies. These accumulate in the cytoplasmic space of dopaminergic neurons in the *substantia nigra pars compacta* region of the brain, causing cell death, resulting in decreased dopamine levels, and ultimately PD symptoms. To date, a significant proportion of structural information has arisen from in vitro studies using recombinantly purified forms of the protein, often failing to acknowledge that αS is natively located in the presence of phospholipids, where it likely plays a direct role in regulating synaptic vesicle function and neurotransmission. Here we present a series of macromolecular αS assemblies not previously described that form in the presence of lipid vesicles. These fibrillar structures are striking in both their large size relative to those previously reported and by their varying helical content, from ribbons to wave-like helices of long pitch shortening to those more compact and bulkier. These studies provide the foundation for more detailed structural analysis, and may offer new possibilities to further define disease-relevant versions of the protein that are accessible to pharmacological intervention.

## Introduction

α-Synuclein (αS), which has been described as intrinsically disordered or helical, is capable, under certain conditions, of aggregating into a range of different amyloid fibril morphologies^1^. These structures are typically probed via imaging techniques that include electron microscopy (EM) and atomic force microscopy, and more recently at the atomic level using solid-state nuclear magnetic resonance^2^, and increasingly, cryoEM^3–8^. Many approaches describe structures gained via recombinant means under in vitro conditions^2–5,7,9,10^. Others have employed the use of lipids during in vitro experiments, since this is critical to αS function in situ^6,11–17^. More recent studies are emerging in which aggregates of αS from specific neurodegenerative diseases and key regions of the brain are isolated in small quantities^8,18^. The latter have then been used to seed recombinant forms of αS produced in bacteria or cell culture, with the assumption that seed conformation is maintained in mature fibres, without polymorphic shift, and thus those observed within imaging is of the disease-relevant type. To date, all but one of the structures are defined by a double protofilament. Most, but not all, display rotational symmetry about the axis of the fibril.

In particular, the bacterial approach has been used to describe an increasing number of fibril morphologies. Using this approach the first morphologies were described—type 1a (‘rods’^3,5^) and 1b (‘twisters’^5^), as well as types 2a and 2b^9^. This approach has also extended to structures of early-onset αS mutants, including E46K^5,19^ and H50Q^19^. Studying αS using seeds isolated from brain samples and growing mature fibres from them using additional protein produced by bacterial/mammalian cells is also beginning to yield new structures^18^. For example, in multiple system atrophy (MSA) two further unique morphologies (named Type I and Type II) have been described; these contain non-proteinaceous, potentially negatively charged molecules, at the protofilament interface. Interestingly, in both structures each protofibril within the mature fibril adopts a different morphology, rendering the fibrils asymmetric^8^. In contrast to MSA-derived filaments, those seeded from αS isolated from individuals with dementia with Lewy bodies were reported to lack any twist and were reported to be thinner than those from the brains of individuals with MSA, suggested another currently uncharacterised morphological fibril type.

All structures identified to date display a mature fibril width of ~10 nm and a typical helical pitch in the Angstrom range (e.g. ~460 Å for twisters, or ~920 Å for rods). Here we describe very different and much larger assemblies that have not been previously reported, and which form specifically in the presence of lipid vesicles. This is particularly interesting given the emerging discussion over whether Parkinson’s disease (PD) is a proteinopathy or lipidopathy^20^. These newly reported structures are able to be readily viewed using transmission EM (TEM) and range in polymorph from structures that are ribbon like to those that are highly helical and compact, with a range of observed helical assemblies in between. Interestingly, the addition of lipid vesicles to the monomeric αS sample appears to be an absolute requirement for the morphological shift to such larger assemblies. We believe, given the role that αS plays within the cell, that these structures could have biological or pathological relevance and therefore far-reaching implications in defining a disease-relevant conformation towards identification of a druggable target.

## Results and discussion

### Lipid vesicle-induced fibril polymorphs

The addition of lipids to αS has been reported to modulate misfolding and toxicity^21^, causing the protein to form an extended helical structure on small unilamellar vesicles (SUVs). The binding of αS to lipid membranes has complex effects on the latter^13^, altering the curvature of the bilayer structure and leading to the formation of small vesicles^14^. αS has been shown to be capable of bending membranes consisting of negatively charged phospholipid vesicles and forming tubules from large lipid vesicles^22^.

**Fig. 1:**
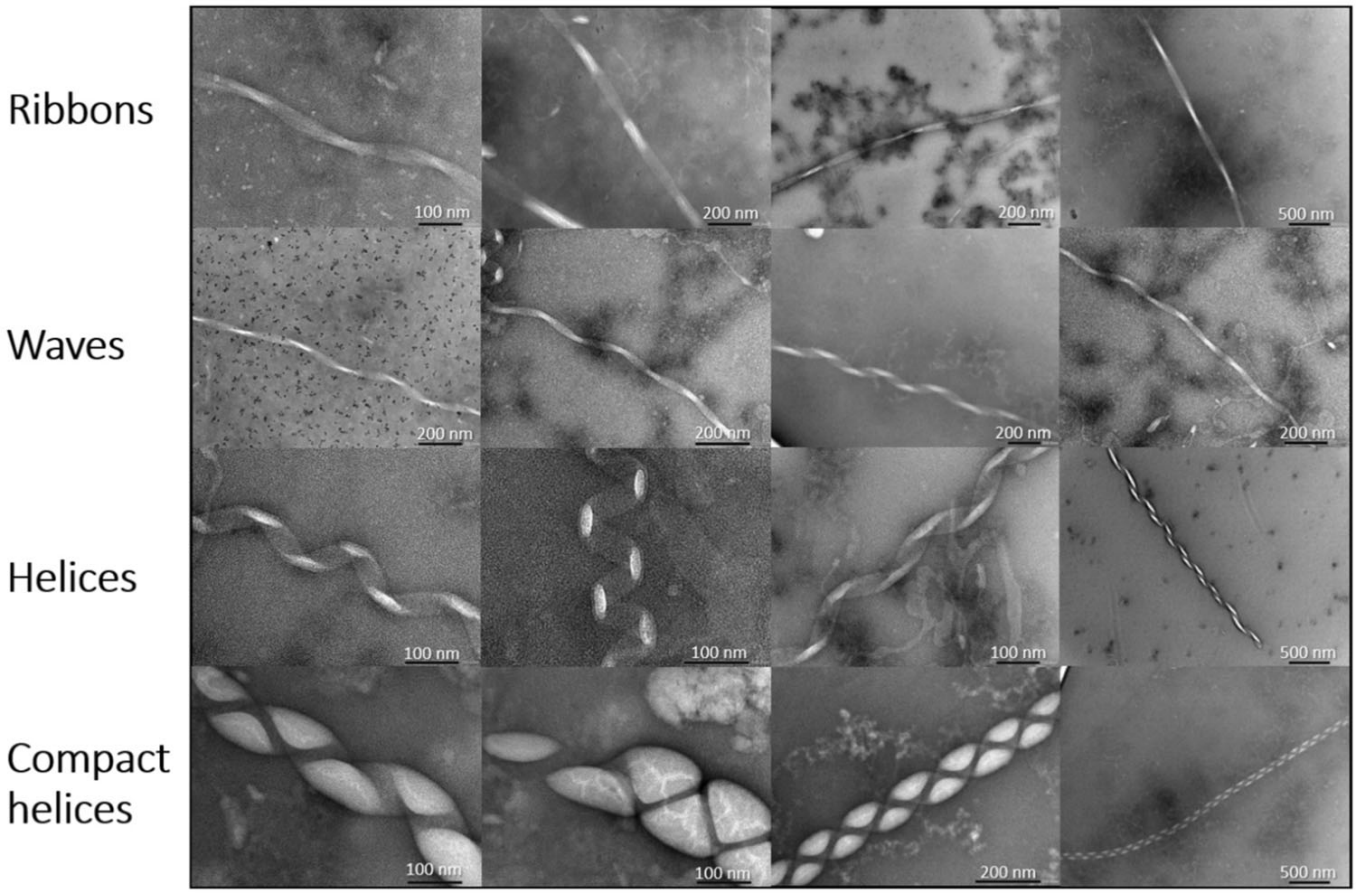
A variety of large fibril polymorphs are formed upon incubation of αS with DMPS lipid vesicles. These structures were formed by aggregating 100 μM αS in the presence of 200 μM DMPS lipid vesicles at 30 °C for 190 h. The structures have initially been divided into four subtypes with varying degrees of helicity named ribbons, waves, helices and compact helices.

Using TEM we report for the first time the formation of a range of large macromolecular structures that vary widely in their morphology (Fig. 1). In particular, we describe the formation of a much larger type of macro-assembly that can form in the presence of lipid vesicles. Specifically, 1,2-dimyristoyl-*sn*-glycero-3-phospho-l-serine (DMPS) was used to create SUVs, at neutral pH (pH 6.5, 30 °C), as previously described^15,23,24^. DMPS vesicles were chosen as a model phospholipid system since they are a key component within dopaminergic synaptic vesicles, and the negative charge on their surface has been shown to be capable of significantly promoting αS primary nucleation^15,16^. Interestingly, DMPS lipid vesicles have been previously shown to act by forming kinetically trapped αS protofibrils^25^. Moreover, lipid-induced fibril production has been shown to be strongly affected by early-onset mutations, which can induce dramatic changes in such crucial processes thought to be associated with the initiation and spreading of αS aggregation^26^. Phospholipid biosynthetic enzymes have also been seen to be elevated in the substantia nigra of PD patients^27^. Specific membrane interactions can therefore play a key role in triggering a conversion of αS from a soluble to aggregated form that is associated with disease.

**Fig. 2:**
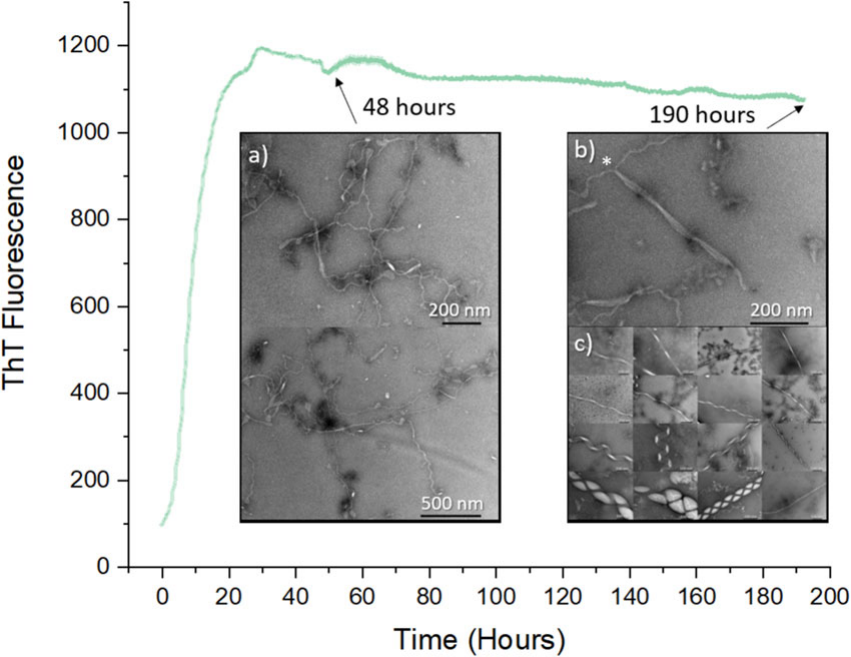
Lipid-induced αS aggregation followed by ThT fluorescence. Aggregation of 100 μM αS in the presence of 200 μM DMPS lipid vesicles at 30 °C, followed by ThT fluorescence and TEM. **a** TEM samples taken at 48 h show the formation of meandering fibril-like structures growing from the surface of punctate lipid vesicles. These are observed to be ~5 nm in width and straight, or ~10 nm in width (Supplementary Fig. 3). **b** TEM samples taken at ≈190 h show two meandering 10 nm fibrils fusing to form a 40-nm-wide ribbon-like structure*. **c** TEM samples observed at ≈190 h display a range of macromolecular polymorphs (image is shown in more detail in Fig. 1).

To induce the formation of the macromolecular structures, briefly, 100 μM monomeric αS (Supplementary Fig. 1) was mixed with 200 μM DMPS SUVs, of ~30–40 nm diameter (as measured by dynamic light scattering (DLS); Supplementary Fig. 2), under quiescent conditions (Fig. 2). The solution was left to aggregate, as monitored by ThT fluorescence, for a period of 48 h, allowing the fluorescence to progress through the lag and exponential phases of aggregation before plateauing at this time at the stationary phase. A sample was collected from this phase and analysed by TEM (Fig. 2a). During this time distinctive fibril-like structures were observed to emerge from the surface of the SUVs that are similar to those reported by others in the presence of DMPS^6,15^. In particular, these meandering fibril-like structures, were ~5 nm in diameter, suggesting that they are protofibrils of the single-stranded variety^2^. Also observed were wider fibrillar structures, measuring 10 nm in diameter (Supplementary Fig. 3), suggestive of a more mature double strand fibril structure that is consistent with previously reported dimensions^3–5^. The samples were next left to further incubate at 30 °C until 190 h had passed, upon which the helical assemblies reported were observed. Since lipid-induced meandering protofibrils may represent kinetically trapped intermediates^6,25^, extended incubation times may be required for conversion to the much larger helical assemblies reported here. Of note during this period is the fact that no detectable change in ThT fluorescence was observed (Fig. 2; Supplementary Fig. 4). This may explain why these structures have not been previously reported, since it also suggests that no additional ThT becomes bound during the formation of these structures. At 190 h the sample was again analysed by TEM, with fibril-like structures appearing to aggregate into much larger macromolecular assemblies (Fig. 2b). Within the sample a range of macromolecular polymorphs not previously described can be seen ranging in width and helicity (Figs. 1, 2b).

**Fig. 3:**
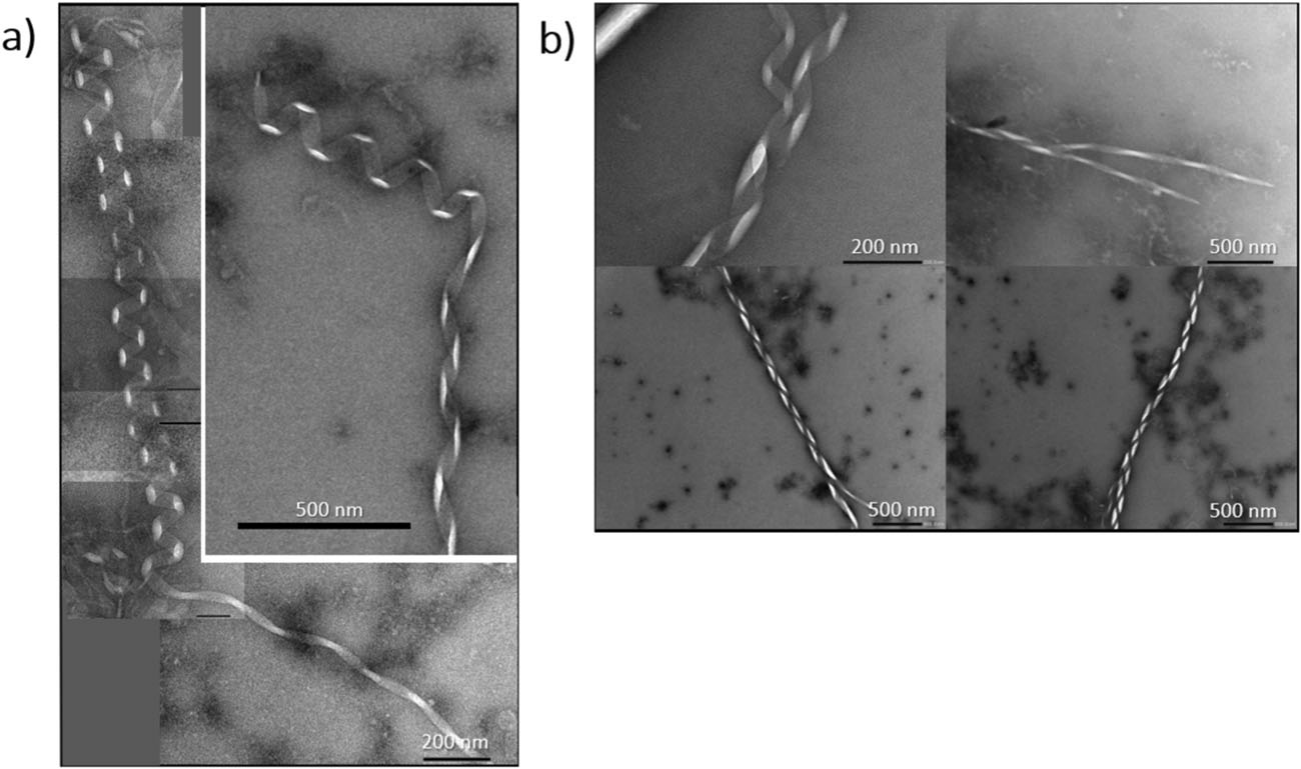
Polymorph unwinding. **a** αS polymorph ‘helices’ are observed to unwind into ‘ribbons’ and ‘waves’. **b** The αS macromolecular assemblies have been observed to have frayed ends, suggesting a common building block between the different polymorphs.

The width of the assemblies are up to ten times larger than αS structures reported previously; up to 100 nm, with helical pitches of ~40–100 nm (Fig. 1). Observed helicity is also wide ranging; in the most elongated assemblies (Fig. 1), we observe ribbon-like structures (‘ribbons’) with a pitch ~200 nm, increasing to low helicity structures (‘waves’) with a similar pitch of ~200 nm. Next, we observe structures that resemble a classic helical signature (’helices’) with a pitch of ~100 nm. Finally, very electron-dense compact helical structures (‘compact helices’) are formed. ‘Ribbons’, ‘waves’ and ‘helices’ are all ~40–50 nm wide, but as would be expected, ‘compact helices’ by contrast are much larger at ~100 nm in width. All structures are capable of reaching many microns in length with some variations in width and helicity. Interestingly, we also observe changes in helical type within the same fibril, suggesting that ‘helices’, ‘waves’ and ‘ribbons’ are interconvertible (Fig. 3a). Finally, we also report upon branched fibril termini, which may represent the formation of the larger assemblies from smaller structures (Fig. 3b). This also suggests that these macromolecular assemblies may be formed by the same common αS fibril building blocks that then cluster adjacently along the vertical axis. Reassuringly, the same structures were observed upon three separate repetitions of the experiment. These were undertaken weeks apart and in each case were prepared using both fresh αS and DMPS vesicle preparations (Supplementary Fig. 5), demonstrating both the reproducibility of our findings and similar morphologies of the αS aggregates observed (Supplementary Fig. 6).

Regarding molecular determinants of αS–lipid interaction that may predetermine the formation of the structures we report, it has been proposed that lysine-rich positions within αS may interact with negatively charged lipid head groups, while hydrophobic regions may interact with membrane lipids, leading to an initial helical conformation^1,28,29^. Specifically, the central region of the protein (residues 26–97) has been shown to bind to lipid membranes and positive charge within the N-terminal region may also play a key role in mediating binding to anionic lipids^30,31^. A high local concentration of αS at the membrane could potentially lead to primary nucleation and a conversion to β-sheet formation. The position of single amino acid changes associated with early-onset PD mutations is likely to alter sidechain–sidechain or sidechain–lipid interactions that modulate helix formation, thereby accelerating the pathway toward amyloidosis^26^.

Given the proposed native role of αS in synaptic transmission and signalling, and the clear interaction of αS with dopaminergic neuronal membranes, the discovery of these structures in the presence of phospholipid vesicles is likely to have far-reaching implications towards defining a pathogenic αS conformation that has significant potential to open up new routes towards new conformations and drug targets for diseases in which αS aggregates present.

## Methods

### Protein expression and purification of human wild-type αS

Wild-type (wt) human αS was recombinantly expressed and purified based on, and modified from, a previously published method (Supplementary Fig. 1)^32,33^. Briefly, the pET21a plasmid containing the human wt αS (1–140), purchased from Addgene (deposited by the Michal J. Fox Foundation (MJFF), Plasmid #51486), was transformed into *Escherichia coli* expression cell line BL21 (DE3). 2xYT overnight cultures containing ampicillin of this human wt αS (1–140) pET21a BL21 (DE3) *E. coli* strain were used to inoculate 1 l 2xYT cultures, containing 100 mg l^−1^ ampicillin, and grown at 37 °C, 200 r.p.m. shaking, to OD_600_ = 0.6–0.8 and induced with 1 mM isopropyl-1-thio-d-galactopyranoside at 37 °C, 200 r.p.m. shaking, for 4 h in an Innova 44 Incubator shaker (New Brunswick Scientific). The bacteria were harvested by centrifugation at 4600 × *g*, and resuspended in 40 ml of 20 mM Tris buffer pH 8 containing 1 complete protease inhibitor tablet (Roche) and freeze-thawed at −20 °C before lysis, by sonication. The cell debris was discarded by centrifugation at 48,400 × *g*, and the supernatant was collected and boiled at 95 °C for 10 min. The precipitated protein was removed by centrifugation at 18,500 × *g*. The supernatant was collected, and ammonium sulfate was added to 30% saturation (0.176 g ml^−1^), and left shaking at 20 °C for 1 h. The precipitated protein, containing the αS, was harvested by centrifugation at 18,500 × *g*, and then resuspended in 50 ml of 20 mM Tris buffer pH 8 by gentle agitation at 4 °C. The proteins were purified by anion exchange chromatography on an AKTA pure purification system (GE Healthcare) with a 5 ml HiTrap Q HP (GE Healthcare) pre-packed column, to remove protein impurities and protein-bound nucleic acids. The purified fractions were combined and further purified by size-exclusion chromatography (SEC), using a HiLoad 16/60 Superdex 75 pg (GE Healthcare) pre-packed purification column, to buffer exchange the αS into the relevant reaction buffer (20 mM sodium phosphate buffer pH 6.5/20 mM sodium acetate pH 5.0) and ensure that only monomers were collected. Pure monomeric αS was eluted between 54 and 60 ml.

The concentration of the purified αS was determined in a 2-mm path length quarts cuvette, using an extinction coefficient of 4836 M^−1^ cm^−1^ at 280 nm, separated into 1 ml aliquots, snap frozen in liquid N_2_, and stored at −80 °C until required.

The purity of αS following SEC was confirmed by sodium dodecyl sulfate-polyacrylamide gel electrophoresis, and the correct mass was confirmed by mass spectrometry on a Dionex Acclaim RSLC Polar Advantage II (PA2), 2.2 µm, 120 Å, 2.1 × 50 mm^2^ (Thermo Fisher Scientific, California, USA). The deconvoluted average mass of the protein was confirmed as 14,459.749*m*/*z*, representing the mass of wt human αS (1–140). A circular dichroism spectra scan was performed, to confirm the random coil conformation of the monomeric αS stock (Supplementary Fig. 5).

### Microplate ThT kinetic assays without shaking to measure lipid-induced aggregation

ThT kinetic assays for lipid induced primary nucleation of αS were performed in a CLARIOstar fluorescence microplate reader (BMG Labtech), under quiescent conditions (without shaking), 30 °C in black, clear-bottomed 96-well half area polystyrene plates with non-bonding surface (Corning #3881) covered with Aluminium Thermowell Sealing Tape (Corning #6570). The focal height was set to 4.9 mm, and gain to 800, with an excitation filter of 440–15 nm and emission filter of 480–15 nm and a dichroic cut-off of 460 nm. Well measurements were taken by spiral average of 4 mm using the bottom optic, with 50 flashes per well and a cycle time of 1200 s. The outer wells of the plate were not used. The experiments were performed in 100 μl aliquots, in triplicate, each containing 100 μM αS, 50 μM ThT, 200 μM DMPS, and 0.01% sodium azide in 20 mM phosphate buffer pH 6.5.

### Lipid preparation for induced primary nucleation method

The mass of dry DMPS lipid powder was determined using an ultra-micro balance (Sartorius), and dissolved in 20 mM sodium phosphate buffer pH 6.5 to a concentration of 2 mM. This was dissolved by shaking, in a 2 ml Eppendorf tube, on a Thermomixer compact (Eppendorf), at 45 °C, 1400 r.p.m. for 3 h. The solution was then freeze-thawed five times using dry ice and the thermomixer compact (Eppendorf) at 45 °C and 500 r.p.m. The preparation of the vesicles was carried out by sonication, using a Soniprep 150 plus sonicator, set to an amplitude of 10.0, for five cycles of 30 s on and 30 s off.

### DLS measurements

A sample of the vesicles produced at each step was diluted to 100 μM in 20 mM phosphate buffer pH 6.5. Their size distribution was measured by DLS, using a Zetasizer Nano ZSP (Malvern Instruments), to ensure a final consistent size of between 20 and 30 nm was obtained (Supplementary Fig. 2).

### Transmission electron microscopy

αS samples from the end point of the aggregation kinetics were collected. Five microlitres of these samples were put onto glow-discharged Formvar/carbon-coated, 200-mesh, copper grids for 1 min. The samples were dried with a filter paper, and then washed twice with Milli-Q water for 1 s, and removed each time with filter paper. The sample was stained by incubating the grids with 5 µl Uranyl Acetate Zero (Agar Scientific) for 30 s, followed by removal of the excess stain with a filter paper. The grids were left to air dry for 2 h. The samples were imaged using a Transmission Electron Microscopy Jeol 2100 Plus (JEOL), operating at an accelerating voltage of 200 kV. Multiple grids were screened in order to obtain representative images of the samples.

### Reporting summary

Further information on experimental design is available in the Nature Research Reporting Summary linked to this article.

## Supplementary information

Supporting Informtion

Reporting Summary

## Data Availability

The datasets generated during and/or analysed during the current study are available from the corresponding author on reasonable request.
