# Supplementary material for: A series of helical α-synuclein fibril polymorphs are populated in the presence of lipid vesicles
Source: NPJ Parkinsons Dis. 2020 Aug 19;6:17. doi: 10.1038/s41531-020-00122-1 (PMC7438318; doi:10.1038/s41531-020-00122-1)
Supplement: Supplementary file 1 — Supporting Informtion [file 41531_2020_122_MOESM1_ESM.pdf]

Supporting Information

Production and of Purification of Human wt αS

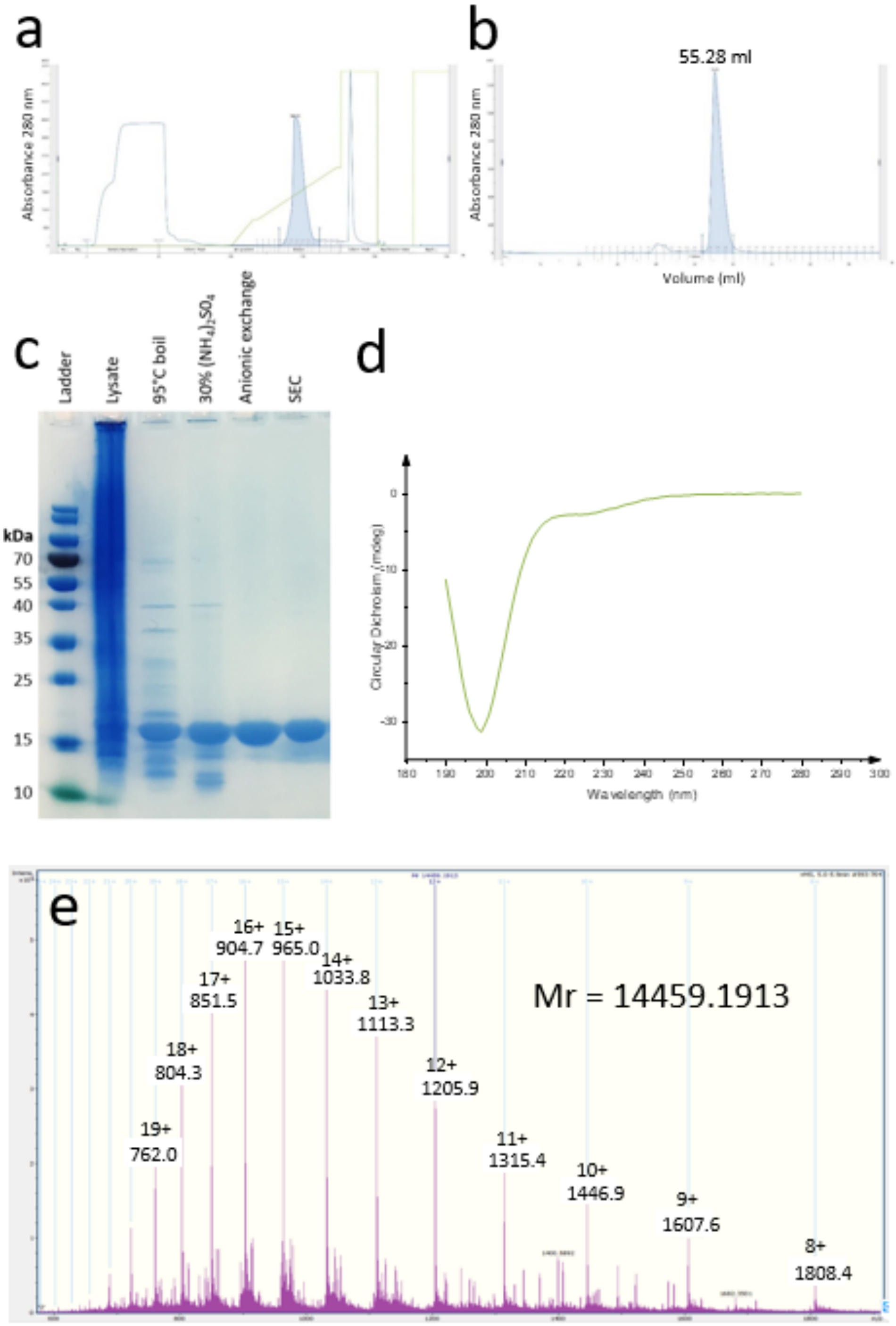

Supplementary Figure 1: Overview of the purification of αS used for the experiments. a) Chromatograph of the Hi-Q anionic exchange purification. b) Chromatograph of the Size exclusion chromatography and buffer exchange. c) SDS page gel showing an overview of the entire purification protocol. d) Far-UV circular dichroism spectra of the purified αS showing that the monomeric αS is in a random coil conformation. e) De-convoluted mass spectrum, showing a mass of the protein of 14459 m/z, representing the mass of wt Human αS (1-140).

Production and of DMPS SUVs

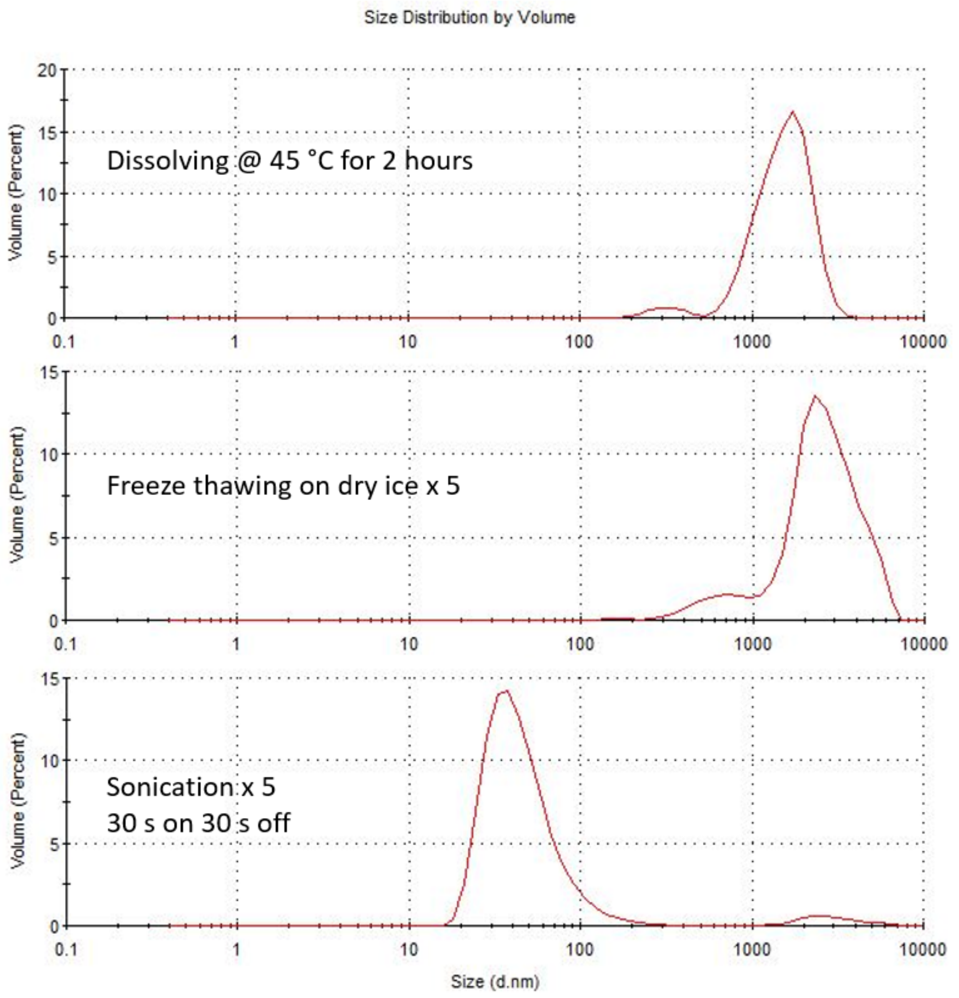

Supplementary Figure 2: Dynamic light scattering size distribution of the DMPS small unilamellar vesicles (SUVs) used for lipid induced nucleation assays, showing a size distribution centred around 30 nm post sonication.

Production and of DMPS SUVs

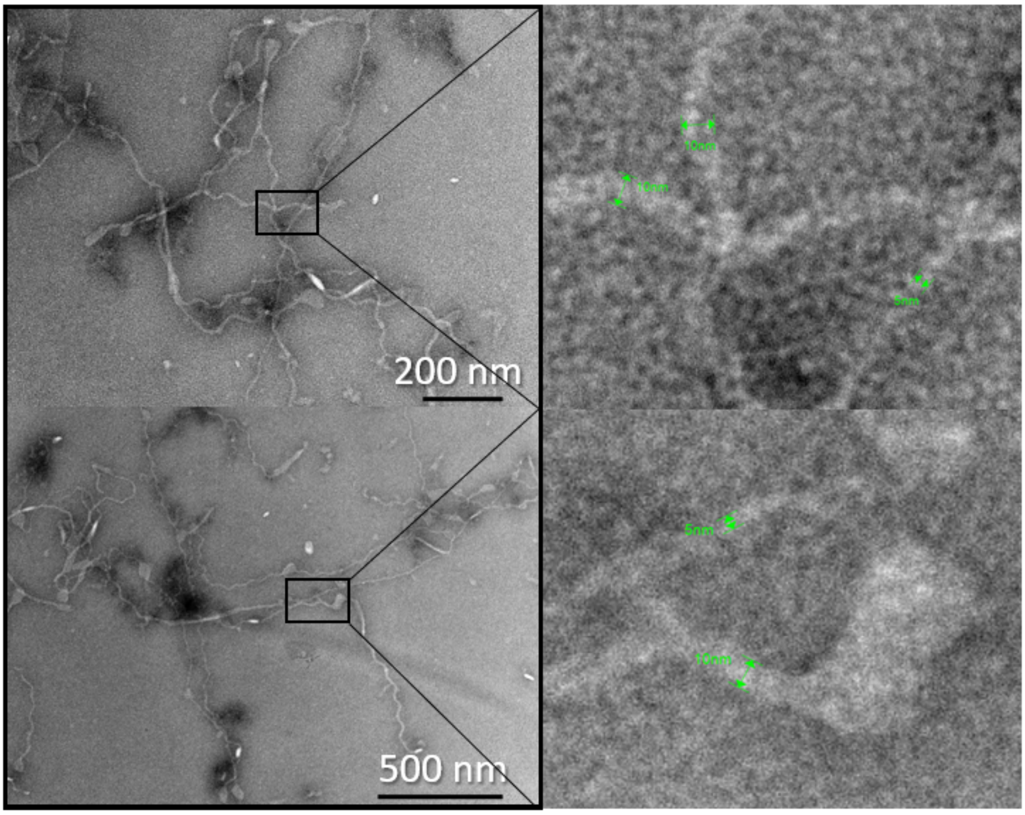

Supplementary Figure 3: TEM images of fibril like structures from 48 hours incubation with lipids, displaying fibril widths of 5 and 10 nm width wavy fibrils.

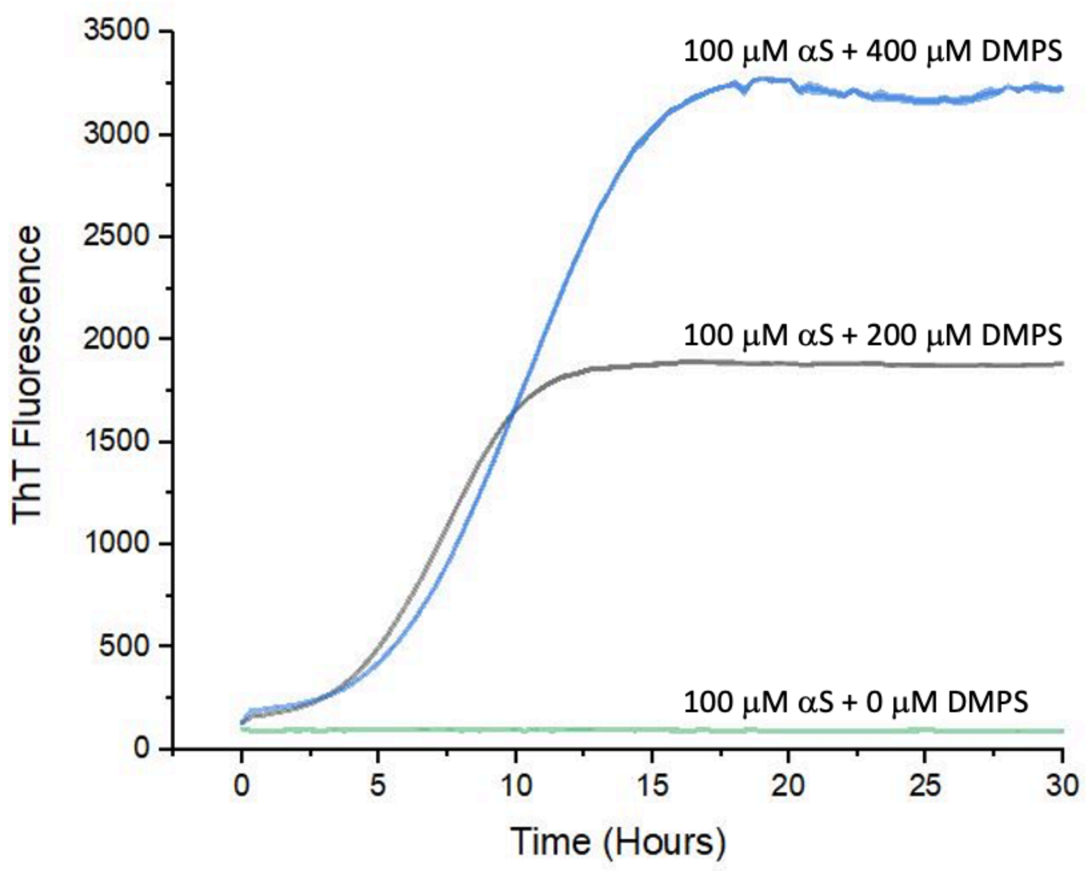

Supplementary Figure 4: αS incubation in the presence and absence of DMPS SUVs. αS (100 μM) in 20 mM sodium phosphate buffer (pH 6.5), with ThT (50 μM), incubated with DMPS (200 μM – grey; 400 μM - blue), or in the absence of DMPS (green) at 30 °C under quiescent conditions Each trace is the average of three repeats showing standard error.

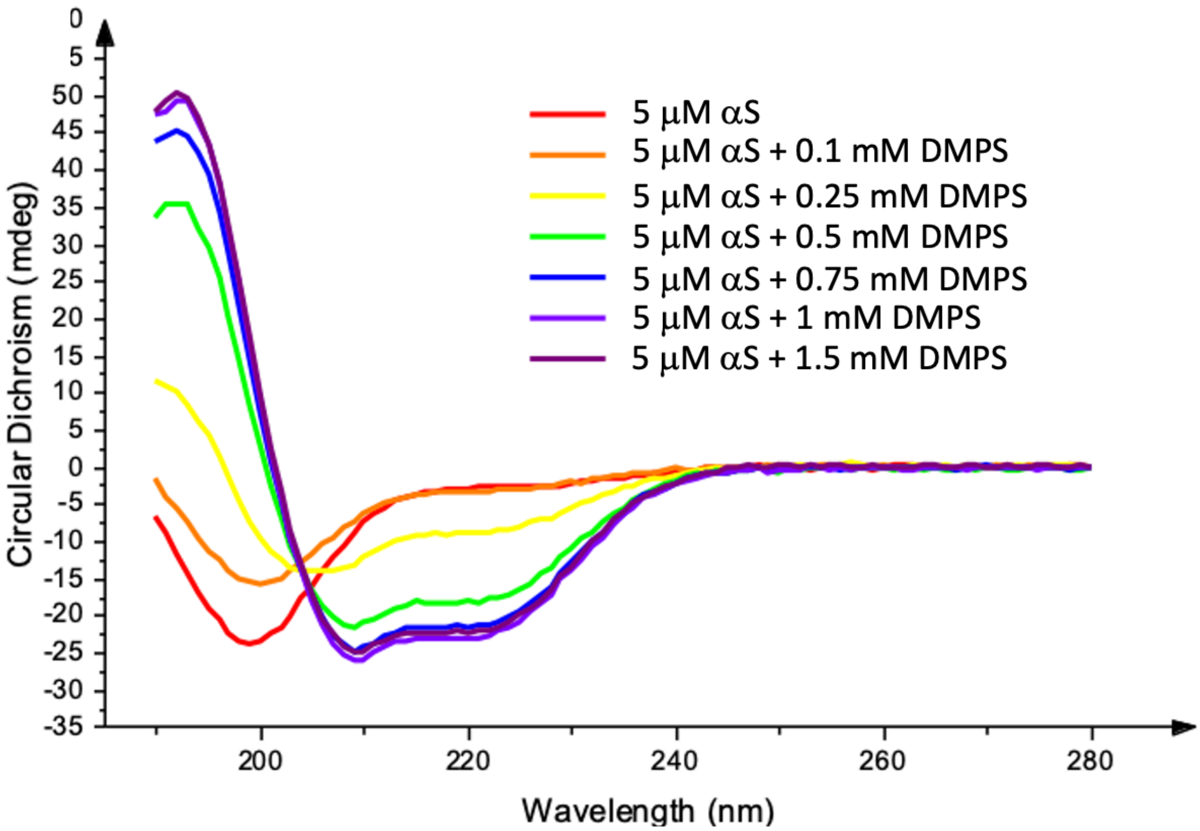

Supplementary Figure 5: Circular Dichroism studies in the presence of lipid vesicles *a)* Circular dichroism of αS with increasing concentrations of DMPS SUVs. In isolation αS exists as a random coil. The conformation of αS shifts towards an α-helical structure with increasing concentration of DMPS SUVs. Data presented represent an average of three repeats.

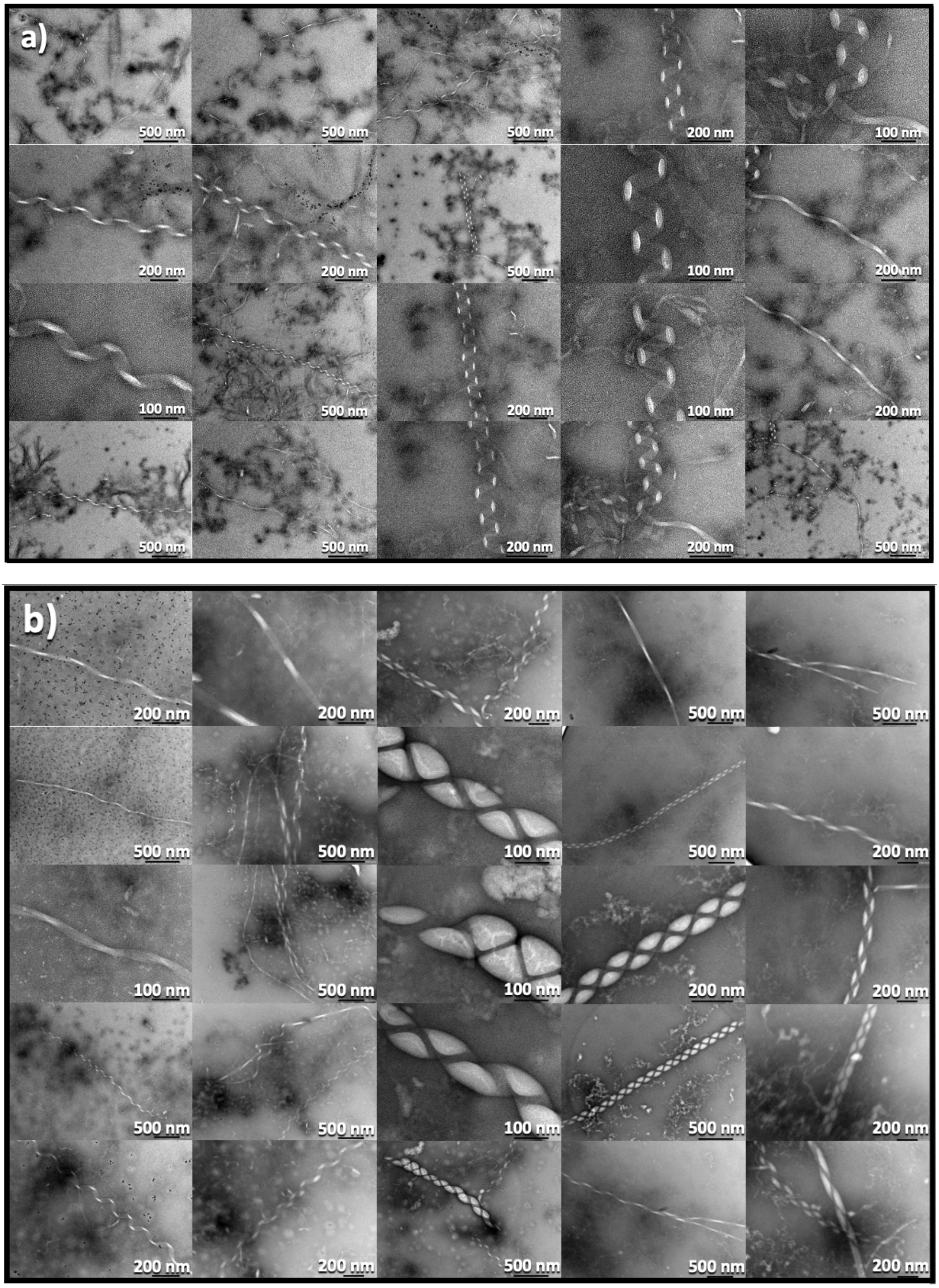
a)
500 nm
500 nm
500 nm
200 nm
100 nm
200 nm
200 nm
500 nm
100 nm
200 nm
100 nm
500 nm
200 nm
100 nm
200 nm
500 nm
500 nm
200 nm
200 nm
500 nm
b)
200 nm
200 nm
200 nm
500 nm
500 nm
500 nm
500 nm
100 nm
500 nm
200 nm
100 nm
500 nm
100 nm
200 nm
200 nm
500 nm
500 nm
100 nm
500 nm
200 nm
200 nm
200 nm
500 nm
500 nm
200 nm

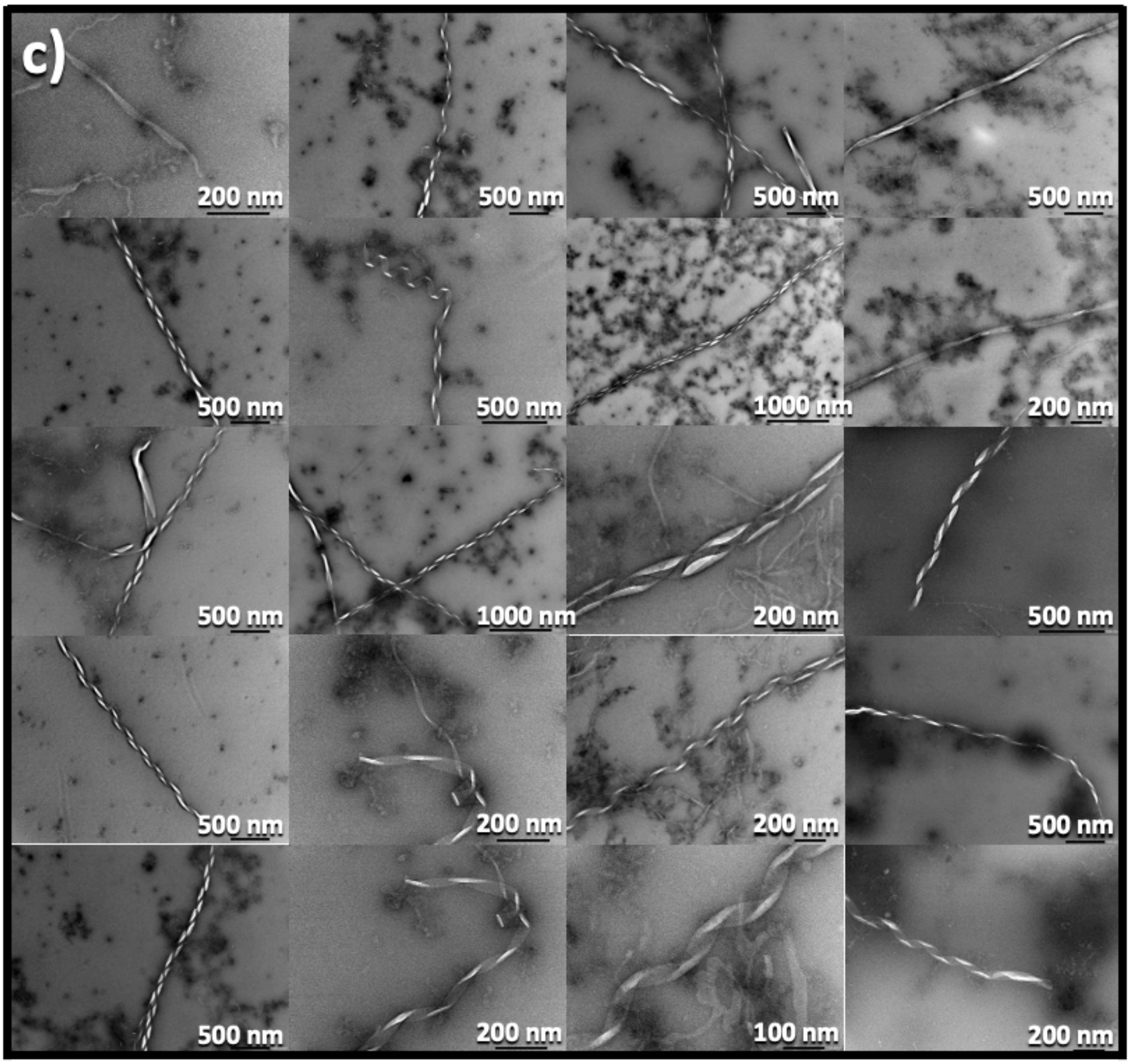

Supplementary Figure 6: TEM images taken from three separate repetitions of the experiment using fresh αS and DMPS SUV preparations. The fibrils were created by aggregating 100 μM αS in the presence of 200 μM DMPS lipid vesicles at 30 °C for 190 hrs. The structures have initially been divided into four subtypes with varying degrees of helicity named ribbons, waves, helices and compact helices (see Fig 1). This work highlights both the reproducibility of the findings and confirms the variability in morphologies of the αS aggregates produced. Shown are fibrils images in *a)* November 2019 *b)* January 2020 *c)* February 2020
